# A New View of the Bacterial Cytosol Environment

**DOI:** 10.1371/journal.pcbi.1002066

**Published:** 2011-06-09

**Authors:** Benjamin P. Cossins, Matthew P. Jacobson, Victor Guallar

**Affiliations:** 1 Department of Life Science, Barcelona Supercomputer Center, Barcelona, Spain; 2 Department of Pharmaceutical Chemistry, University of California San Francisco, San Francisco, California, United States of America; University of Illinois, United States of America

## Abstract

The cytosol is the major environment in all bacterial cells. The true physical and dynamical nature of the cytosol solution is not fully understood and here a modeling approach is applied. Using recent and detailed data on metabolite concentrations, we have created a molecular mechanical model of the prokaryotic cytosol environment of *Escherichia coli*, containing proteins, metabolites and monatomic ions. We use 200 ns molecular dynamics simulations to compute diffusion rates, the extent of contact between molecules and dielectric constants. Large metabolites spend ∼80% of their time in contact with other molecules while small metabolites vary with some only spending 20% of time in contact. Large non-covalently interacting metabolite structures mediated by hydrogen-bonds, ionic and *π* stacking interactions are common and often associate with proteins. Mg^2+^ ions were prominent in NIMS and almost absent free in solution. Κ^+^ is generally not involved in NIMSs and populates the solvent fairly uniformly, hence its important role as an osmolyte. In simulations containing ubiquitin, to represent a protein component, metabolite diffusion was reduced owing to long lasting protein-metabolite interactions. Hence, it is likely that with larger proteins metabolites would diffuse even more slowly. The dielectric constant of these simulations was found to differ from that of pure water only through a large contribution from ubiquitin as metabolite and monatomic ion effects cancel. These findings suggest regions of influence specific to particular proteins affecting metabolite diffusion and electrostatics. Also some proteins may have a higher propensity for associations with metabolites owing to their larger electrostatic fields. We hope that future studies may be able to accurately predict how binding interactions differ in the cytosol relative to dilute aqueous solution.

## Author Summary

The cytosol is the major cellular environment housing the majority of cellular activity. Although the cytosol is an aqueous environment, it contains high concentrations of ions, metabolites, and proteins, making it very different from dilute aqueous solution, which is frequently used for in vitro biochemistry. Recent advances in metabolomics have provided detailed concentration data for metabolites in *E.coli*. We used this information to construct accurate atomistic models of the cytosol solution. We find that, unlike the situation in dilute solutions, most metabolites spend the majority of their time in contact with other metabolites, or in contact with proteins. Furthermore, we find large non-covalently interacting metabolite structures are common and often associated with proteins. The presence of proteins reduced metabolite diffusion owing to long lasting correlations of motion. The dielectric constant of these simulations was found to differ from that of pure water only through a large contribution from proteins as metabolite and monatomic ion effects largely cancel. These findings suggest specific protein spheres of influence affecting metabolite diffusion and the electrostatic environment.

## Introduction

The composition of metabolites, ions and proteins, and processes such as metabolism and signalling which take place in the *E.coli* cytosol are largely well defined [1], [2]. However, the structure and dynamical nature of the cytosol solution is less well understood whether on the local or cytosol-wide levels. Current perception of the cytosol solution is often of a unstructured mixture with behaviour that differs quantitatively but not qualitatively from an ideal solution. Alternatively, there are theoretical descriptions of a cytosol organised into functionally specific regions and even separated protein and small molecule regions linked by metabolite transit pathways [3], [4].

Cytosolic metabolites are extremely varied, but a large majority of these molecules are negatively charged. Assumed electro-neutrality is maintained by a large concentration of potassium ions and to a lesser degree by magnesium and poly-amines such as putrescine and spermidine. The large amount of charge in the cytosol suggests that electrostatics is a dominant force. However, the Debye length at physiological ionic strength is very short (less than 1 nm) [3], [4]. This electrostatic screening is probably essential for the observed extent of macromolecular crowding [5].

The charge distribution and dynamics of the solution also determines the dielectric constant (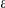), which is reduced by increasing concentration of monatomic ions [6], [7] while Zwitterionic metabolites are thought to increase 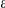 [8]–[10]. The effect of proteins seems to vary, with some studies suggesting an increment [11]–[13] and others a decrement [14], [15]. Experimental data on 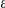 of the cytosol is sparse but in general it suggests that cytosolic 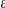 is significantly larger than that for pure water [16]–[19].

The hydrophobic effect is also significantly modulated by ionic strength. Increasing salt concentration increases the strength of the hydrophobic effect [20] possibly through the weakening of water hydrogen bonding [21]. Almost all theoretical treatments of these issues assume simple solutions of monatomic ions and water, sometimes at infinite dilution. There has been little examination of differences in solutions containing positive monatomic ions and larger, negatively charged solutes.

Given the complexity of the cytosol environment it is very difficult to predict the true nature of structure, dynamics and thermodynamics. With a high level of electrostatic screening and heightened hydrophobicity, is it likely that metabolites and proteins engage in significant and long lasting interactions? A recent theoretical study has attempted to make sense of non-ideal behaviour for two component solutions of some common organic molecules [22]. For some mixtures it was shown that activity can actually decrease with increasing concentration, suggesting a high level of non-ideal behavior. Another study found significantly lower thermodynamic activities between *in vivo* like and standard conditions for enzyme-inhibitor assays, again suggesting significant non-ideal behaviour [23].

Using a recent extensive list of metabolites and their concentrations from exponentially growing *E. coli* [24], we have produced two types of atomistic molecular dynamics simulations of a simplified cytosolic model. One included metabolites only and another also included a protein component, for which we used ubiquitin. Although ubiquitin (PDB code 1UBQ) is a eukaryotic protein, it was chosen owing to its small size and large amount of literature dedicated to its study [25]–[27]. Molecular dynamics allowed us to compute several properties of interest, including 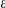, amount of contact between cytosolic molecules and diffusion coefficients. The simulations indicate that metabolites spend a large proportion of their time as part of ‘non-covalently interacting metabolite structures’ (NIMS). Our results also indicate that the cytosolic 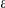 is larger than that of water with monatomic ions. These data allow us to make suggestions about the global structure of the cytosol and the amount of time different metabolites spend free in solution.

## Results

### Structural analysis

This study involved two large cytosol simulations with cubic boxes of 100 Å dimensions, one containing metabolites with monatomic ions (100M) and another with four additional ubiquitin molecules (100MP). Two smaller cytosol simulations (50M and 50MP), a pure water (tip3p) and water + KCl (tip3p+KCl) all with 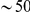 Å dimensions were produced for the dielectric analysis. For a complete list of the simulations of this study and their simplified labels it is instructive to refer to table 1 and the methods section.

**Table 1:** Details of the size and numbers of molecules and atoms in each of four simulations used in this study.

Box	dimension	metabolites	proteins	K+	Mg2+	atoms
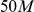	45.4	19	0	22	3	9241
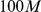	90.85	157	0	175	27	73124
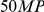	50.0	19	1	22	3	13636
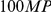	100.0	157	4	175	27	98619
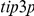	50.0	0	0	0	0	13226
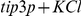	50.0	0	0	26	0	13098

**Figure 1:**
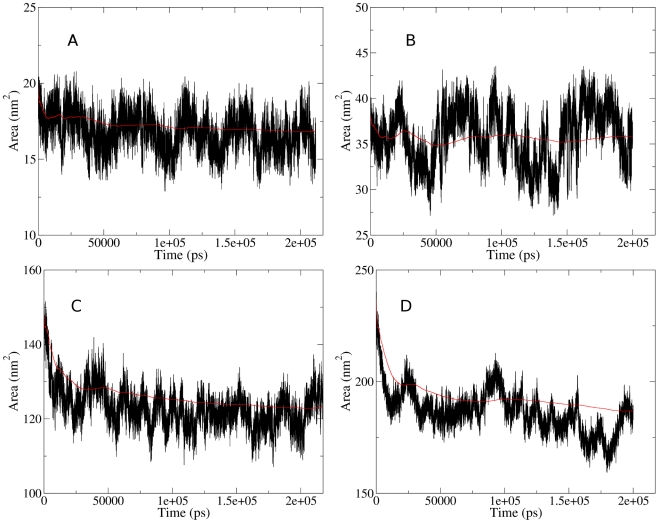
Total SASA of non water molecules, over 200 ns MD simulations of A 50M, B 50MP, C 100M and D 100MP. Red lines are a running average of the SASA data in black.

The structure of cytosol simulations quickly collapsed from almost equal spacing of metabolites to a series of non-covalently interacting metabolite structures (NIMS) inter-spaced with solvent, ions and fully solvated metabolites. This process was conveniently measured through solvent accessible surface area (SASA) of all metabolites except monatomic ions (Figure 1).

Both 50 Å simulations were deemed equilibrated after 30 ns (Figure 1) while the 100M and 100 MP were equilibrated after 35 and 50 ns respectively. Hence, all analyses were carried out only on this structurally equilibrated data (see supporting information Figure S7). Around 16.7% of SASA is lost within the 50 MP system which is similar to the 100 MP box where around 16.4% is lost. These percentage values were calculated using the running averages shown in red in Figure 1. The effect of the box size on metabolite behaviour and general size of NIMS is difficult to gauge but the fact that there is little relative difference between 100 and 50 Å may suggest that smaller box sizes can be used for computationally expensive calculations.

**Figure 2:**
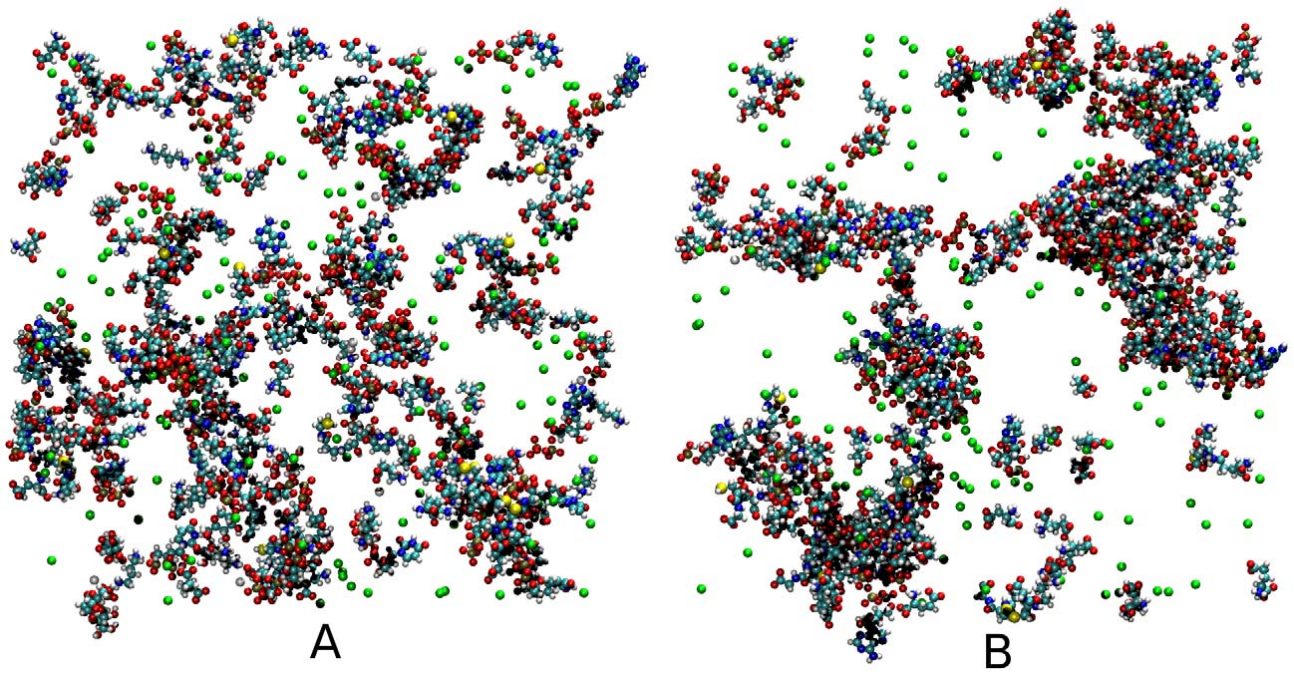
Ball and stick representation of the 100M box at A 0 ns (initial production configuration) and B 200 ns (after structural equilibration). Green and magenta spheres represent potassium and magnesium ions respectively.

**Figure 3:**
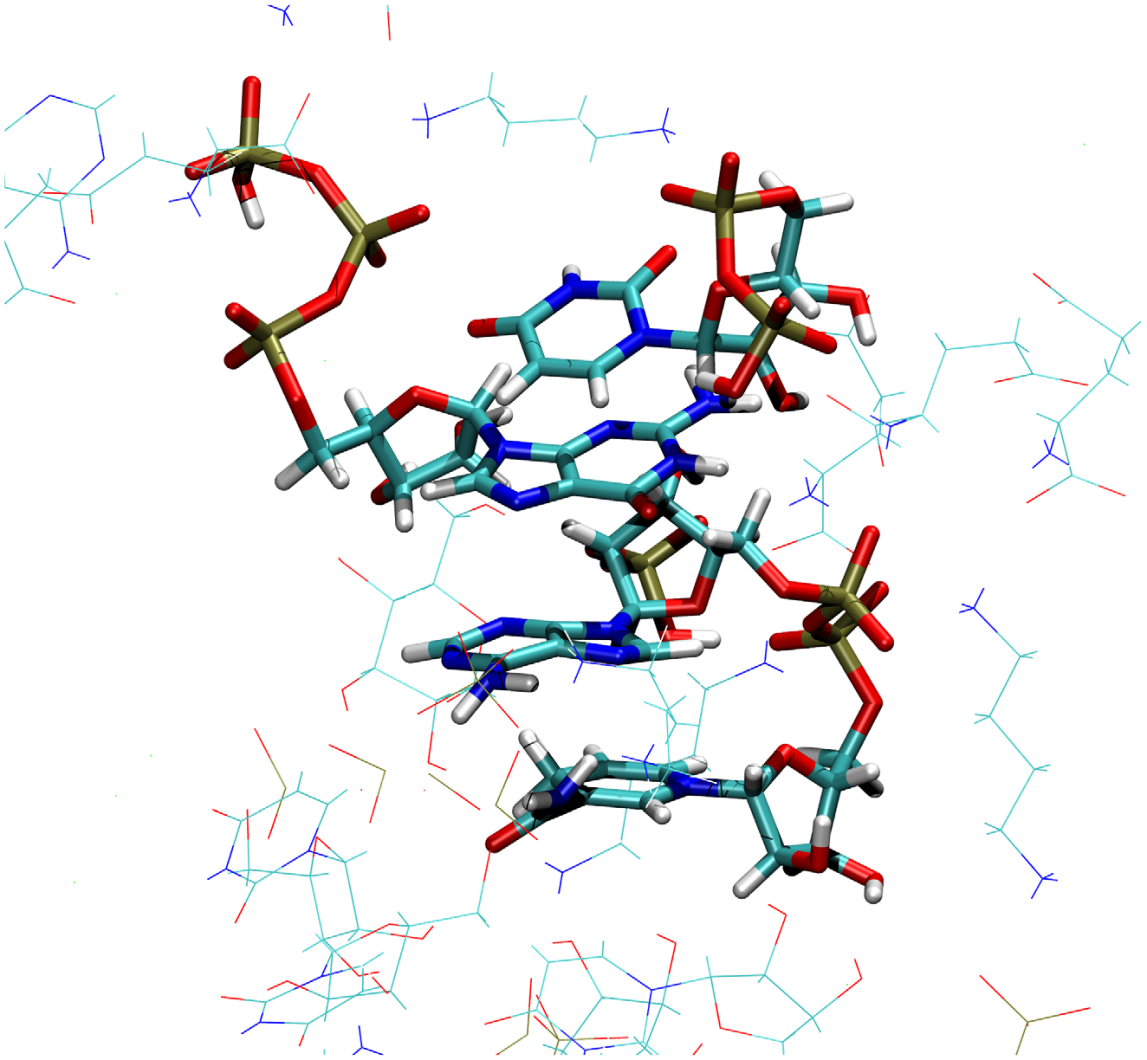
A structure stabilised by 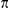 stacking from a 100 Å simulation.

Figure 2 shows a view through the 100 M box at the beginning of the production simulation and after 200 ns. It is clear that after equilibration there is a significant difference in structure. Within the 200 ns simulation of the 100 Å boxes many NIMS were formed which were stable over relatively long time periods. The most interesting of these NIMS were those with a 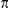 stacking core of nucleotide base like groups (Figure 3 A). These 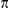 stacks continuously gain and lose bases and persist as long as 50 ns. Some 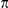 stack NIMS seem reminiscent of RNA and we speculate that these structures often show similarities with the elongation complex of RNA polymerases [28] in the way phosphates are aligned with ribose rings (Figure S3).

**Figure 4:**
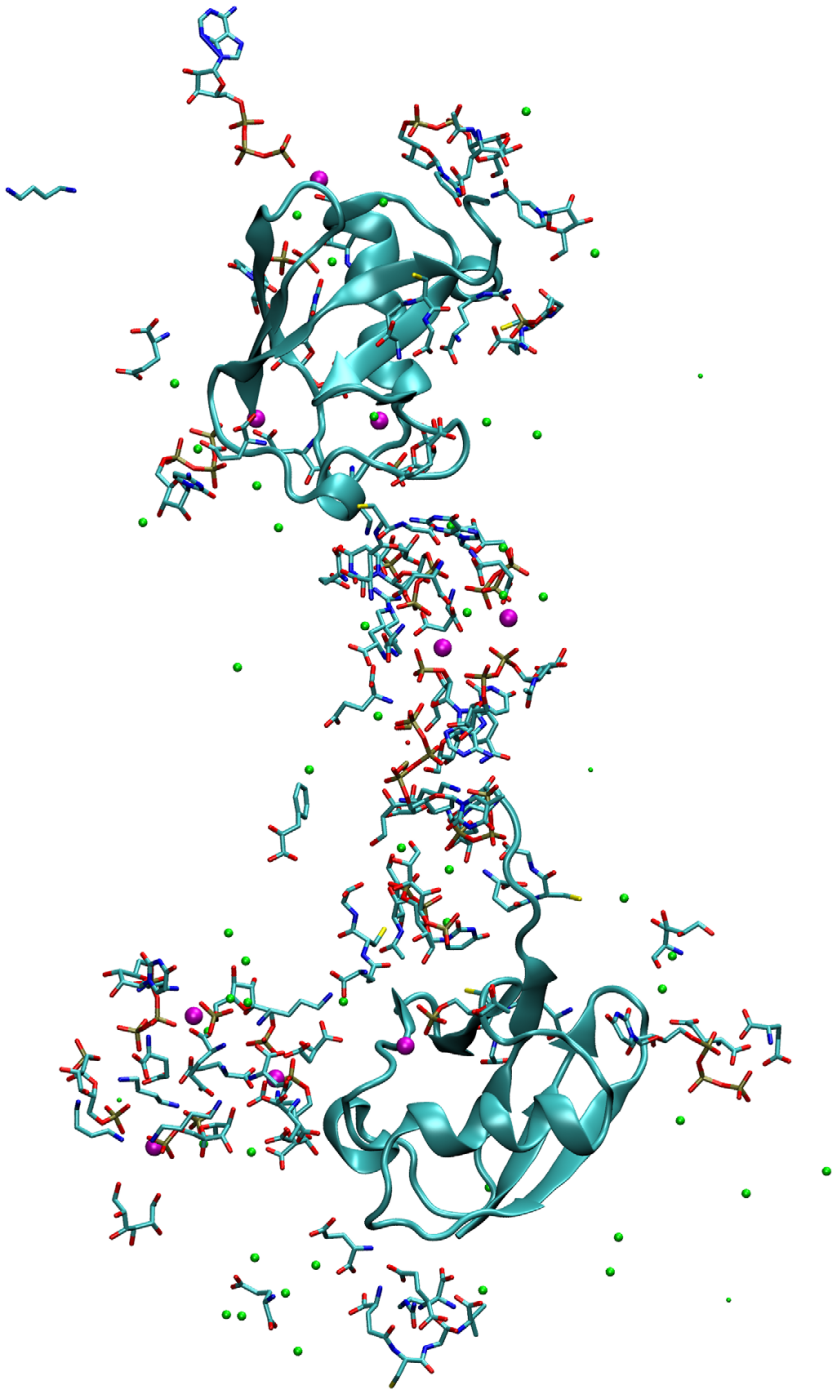
Two ubiquitins with a connecting bridge made up of a NIMS. Mg2+ and K+ ions are represented by large magenta smaller green spheres. Structure was found in the 100 Å simulations.

The inclusion of four ubiquitin molecules perturbed the metabolite structures. Many large NIMSs became attached to protein surface areas containing positively charged residues (Figure 4), in many cases for time periods of 50–100 ns. The attachment or detachment of large NIMS from the protein may contribute to the large SASA fluctuations of Figure 1. These protein-connected NIMSs can also form bridges connecting two proteins which correlates their motions (Figure 4).

### Interactions among metabolites and proteins

SASA analysis was used to investigate any propensity for metabolites to interact. For the SASA and diffusion analyses, only the 100 Å boxes are discussed, however the 50 Å boxes were found to follow similar trends. As might be expected metabolites with larger surface areas have more contact with non-water entities. A comparison of the average contact area in the 100 M and 100 MP boxes for each type of metabolite can be found in the supporting information (Figure S4).

**Figure 5:**
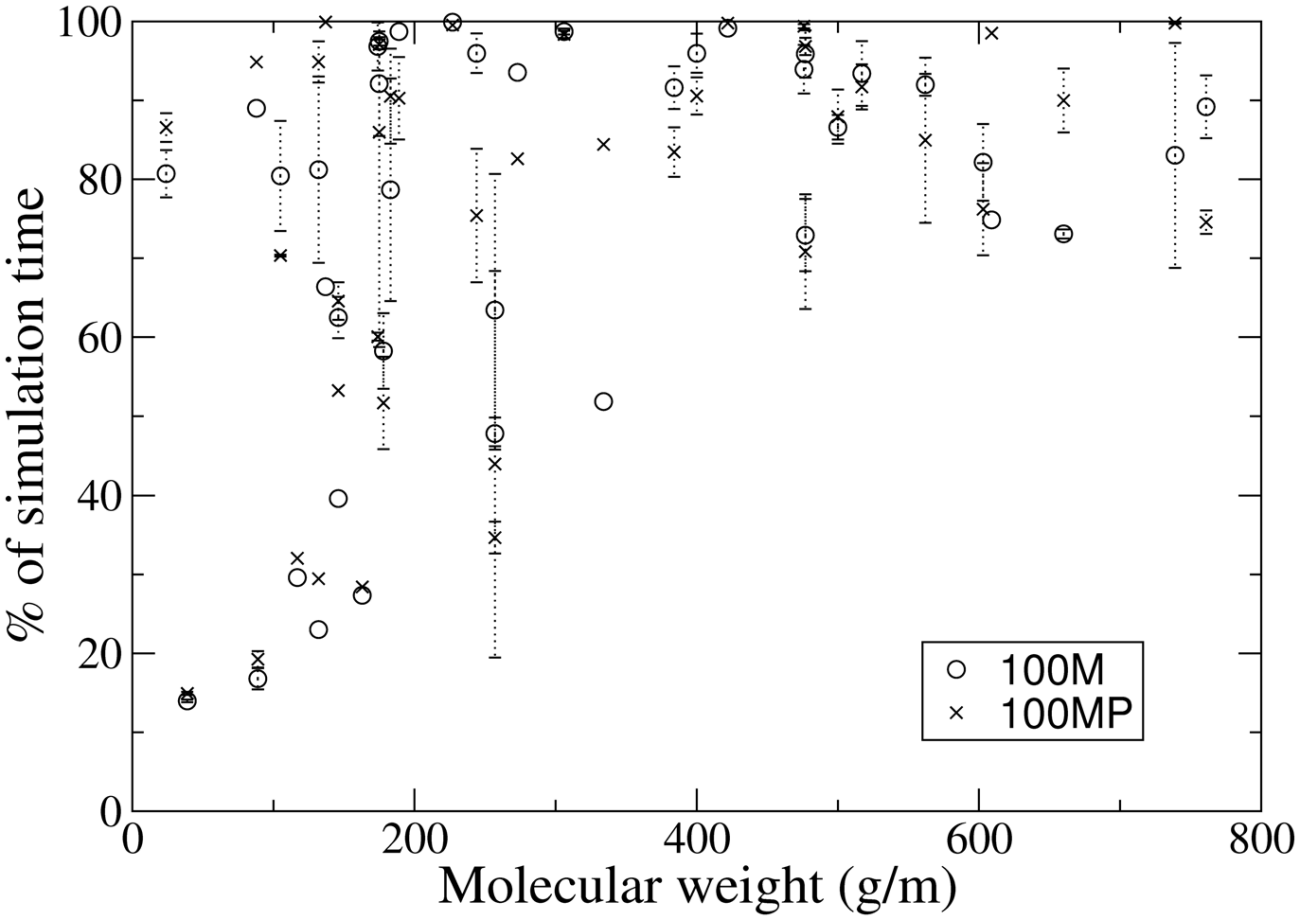
Plot of percentage time which each metabolite is in contact with any another against molecular weight for the 100 Å simulations. Contact is defined through SASA contact area of 0.48 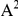 (see methods section). This data is also presented in supporting information Table S1.

Figure 5 displays the amount of time metabolites spend in contact with other molecules and hence are unavailable for any specific interactions. The threshold for our definition of contact is two hydrogen bonds or more (see methods section). Larger metabolites are contacted at least 70% of the time while smaller metabolites show much larger variability with some as low as 20% and other as high as 95%. This analysis gives an indication of metabolites availability for metabolism but of course cannot replace thermodynamic data. A comparison of time in-contact data for the 100 M and 100 MP simulations can be found in supporting information (Figure S5 and Tables S1 and S2). Also further analysis of average and maximum contact events is presented in Figure S2.

A SASA analysis was also applied to ubiquitin, in the 100 MP simulation, to find the metabolite contact area for each ubiquitin residue. Here the residue contact area is defined as the SASA without the environment minus the SASA with the environment and this was reported as a percentage of the average SASA without the environment. Those ubiquitin residues which interact with metabolites most are part of the same patch (nine of the top ten percentage contact area, see supporting information Table S2). Lys 48, becomes covalently attached to the C-terminus of other ubiquitin molecules is part of this patch [25]. This patch was involved in a very close contact event between two ubiquitin molecules in the 100 MP simulation (supporting information Figure S1).

### Diffusion analysis

**Figure 6:**
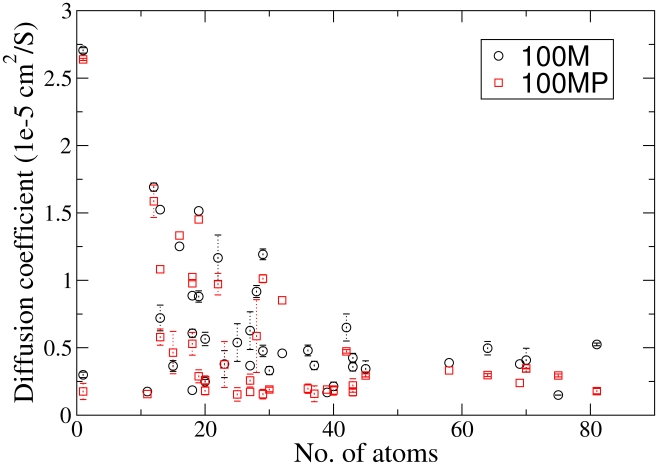
Diffusion coefficients against number of metabolite atoms in the 100 Å simulations. Values were calculated from regression analysis of 100 ps to 2000 ps of a msd plot restarted every 50 ps. Standard errors were calculated from analysis of multiple copies of the same metabolite, metabolites represented only once do not have errors.

**Figure 7:**
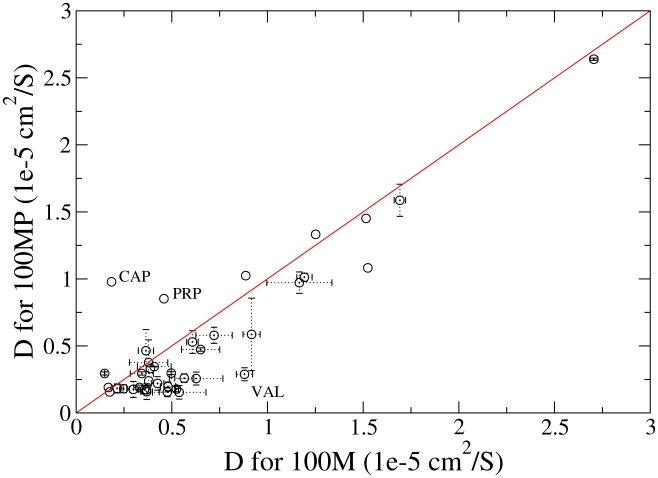
Diffusion coefficients in the without (100M) against with ubiquitin (100MP) simulations. Values were calculated from regression analysis of 100 ps to 2000 ps of a msd plot restarted every 50 ps. Standard errors were calculated from analysis of multiple copies of the same metabolite, metabolites represented only once do not have errors.

Diffusion coefficients were calculated through the Einstein-Helfand relation. Figure 6 shows the diffusion coefficient against the number of atoms for each type of metabolite in the 100 Å boxes. Recent work has shown a periodic box size dependence for water diffusion in water [29]. Here some diffusion rates were slightly reduced in the 50 Å compared to the 100 Å boxes, however many were identical (Figure S6 of supporting information). We have identified only one literature value for metabolite diffusion of 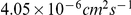 for arginine-phosphate [30], [31], this is within the range of values for molecules with 20 atoms seen in Figure 6. A relation between maximum D and numbers of atoms is clear. However for smaller metabolites (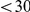 atoms) D ranges over an order of magnitude. It was not possible to find a clear relation between electrostatic charge or hydrophobicity and D. A comparison of D for the 100 M and 100 MP simulations suggesting metabolites diffuse slightly more slowly in the 100 MP simulation is in supporting information (Figure 7).

The diffusion coefficient of ubiquitin in the 100 Å simulation was 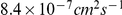, and the average of lateral diffusion in the x, y and z planes was 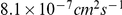. These values can be compared to experimental values for lateral diffusion of 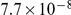 and 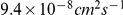 for green fluorescent protein (GFP) in *E.coli* [32]–[35]. The order of magnitude difference in these protein diffusion values can be rationalised by the larger size (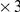) of GFP and the lack of structural proteins and membranes in our simulations. While this comparison is of limited use it is included as this is the most relevent experimental value available and it shows that our computed values are within a reasonable range. Another relevant comparison is with the large Brownian dynamics models of McGuffee *et al.*; here a protein of very similar size (CspC) was found to have a diffusion coefficient of 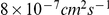 with the smallest observation interval used [36]. In the McGuffee *et al.* study the friction parameter of their Brownian dynamics was adjusted such that the diffusion of green florescent protein matched experimental values. The McGuffee model also differed in that it contained many different types of larger proteins, and so this close agreement may be fortuitous.

### Dielectric constant and conductivity

The dielectric constant (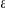) and conductivity (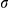) can give insight into the electrostatic properties of a solution and other associated properties such as hydrophobicity. As suggested in the introduction 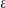 and 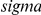 for such a complex heterogeneous solution is difficult to estimate. Owing to the necessity for long simulations with extremely frequent data collection (every 10 fs), smaller simulation boxes were used for this analysis (dimensions of 50 Å). 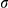 and the translational dielectric constant (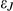) values were found through an Einstein-Helfand analysis described in the theory section. Regression analyses were applied from 100 to 500 ps for all systems except 50 M which used 100 to 300 ps (supporting information Figure S8).

Table 2 shows the results of the present analysis. 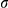 is larger in the simulation without ubiquitin compared to that with ubiquitin, probably owing to the increase in ion and metabolite diffusion (Figure 6). 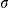 for tip3p water is of course zero, while with the addition of 0.3 M KCL it is greater than the cytosol simulations, caused by higher diffusion rates of charge carriers. The tip3p + KCL 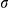 value of 6.69 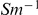 compares well with the experimental value of 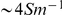 [37].

**Table 2:** Dielectric and conductive properties of cytoplasm, and controls.

Box	50M	50MP	tip3p	tip3p+KCL 0.3M
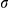	5.1	3.2	0	6.7
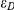	91.5	142.3	92.5	81.9
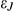	2.5	4.4	0	1.5
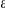	95.0	147.7	93.5	83.8

All dielectric properties are in units of permittivity of free space (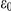) and 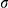 is in 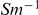. Standard errors for the regression slope (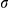) were all less than 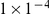. Standard errors for 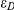 were calculated by block averaging using 10 blocks, all were less than 0.1. Standard errors for the regression intercept (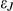) were all less than 0.01. Also 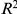 for all regression analyses were higher than 99.

Unfortunately, direct experimental measurements of cytosolic 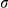 are not available in the literature. However, spherical or spheroidal models (*E. coli* is rod shaped) together with various experimental data have been used to give estimates of *E. coli* cytosolic 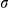. Dielectrophoretic analysis gives 0.35 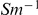 [38], dielectric spectra analysis 0.22 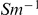 [19] and electrorotation analysis 0.44 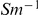 [39]. These model-based measurements also predict a cytosolic 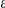 of 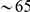, which does not agree with other literature values [16]–[19]. The calculated conductivity with ubiquitin (50 MP) of 3.2 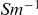 is an order of magnitude greater than these fitted measurements.

Overall, 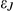 contributions were small compared to total 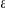. 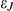 did not relate well to values for 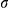 or rates of diffusion. It may be expected that, owing to its large 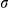, the 50 M system would have the larger 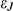 but the 50 MP system contributes far more to 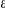 from the conductivity. Also, the tip3p+KCl system has a very small 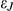 contribution. This suggests a strange difference in the dynamics of charge carriers compared with those in the ubiquitin simulation, vibrating more sharply around a similar position than those in the metabolite only simulation.

The rotational component of 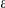, 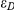, (Table 2) follows trends found in the literature. The pure water system has 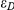 of 92.5 which is slightly lower than some literature calculated values of around 97 [40]. This is almost certainly related to the use of a longer simulation length in this study (data not shown). The tip3p+KCl system had a reduced 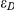 which agrees with another literature study of the SPC water model [6]. The metabolite only system has 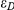 slightly lower than tip3p alone, as the metabolites with large dipoles compensate for the decrementing effect of the salt and those with small dipoles. Finally, the ubiquitin system displays a very large dielectric increment, however, this size of increment is not without precedence [12]. Previous values were similar but used less sampling meaning larger statistical error. Given the relatively small dipole of ubiquitin this increment may be smaller than average.

## Discussion

To the authors knowledge this is the first attempt to produce an atomistic simulation of the cellular cytosol solution. There is relatively little experimental data with which to compare, but comparison with available data on diffusion coefficients was satisfactory.

The 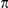 stacking NIMS found here (Figure 3) are interesting and possibly important but are they realistic? Studies comparing aromatic stacking interactions show a reasonable agreement between molecular mechanics free energy calculations, high level electronic structure calculations and experiment [41]–[43]. Also there is experimental evidence for self-association of ATP in solution [44]. However, for guanine-cytosine stacked dimers with and without methyl groups, OPLSAA has been shown to produce non-stacked complexes where other force fields found the correct stacked formation. This may suggest that stacked metabolite complexes could be more prevalent with other force fields [45]. The alignment of phosphate and ribose groups in NIMS, such as that in Figure S3, has similarities to the elongation complex of RNA polymerases and may give an indication of how RNA polymers first emerged. Whilst speculative it is possible that highly reactive conditions (high temperatures or levels of radiation) and large amounts of time could do the job of the catalytic conditions found in RNA polymerases.

The analysis presented here suggests that NIMS are mostly mediated through hydrogen bonds, charge-charge, 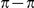 and 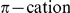 interactions. A recent study has found good agreement in geometries and energies of a large set of relevant intermolecular complexes with high-level *ab initio* calculations [45]. Two other studies have demonstrated the high accuracy of OPLSAA in reproducing association constants of relevent small molecules in chloroform and relative free energies of hydration, heats of vaporization and pure liquid densities for 40 mono- and disubstituted benzenes [43], [46]. No parameter set is perfect but on the whole these study add weight to the idea that the metabolite interactions described here are realistic.

It should be no surprise that 2+ ions are found to be important to metabolite interactions. Many metabolites such as ATP require interaction with 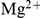 for enzyme-mediated reactions. 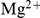 ions were found to have two ionic-bonds or more for more than 80% of both 100 Å simulations (Figure 5). 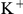 is generally not involved in NIMSs and may populate the solvent fairly uniformly, hence its important role as an osmolyte. All larger metabolites were found to spend 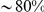 of their time in contact with other molecules. While smaller metabolites vary in diffusive and contact character with some diffusing quickly and maintaining contact only 20–30% of the time. The presence of ubiquitin does not effect the amount of contact time experienced by metabolites.

There is a small difference in diffusion between the two 100 Å systems (Figure 7) which suggests that proteins have an effect on the dynamics of metabolites. In turn this suggests that with larger protein molecules the metabolites diffusion rates would be further reduced. We can speculate that in regions without proteins metabolite diffusion rates would be increased. Recent Brownian dynamics simulations have modeled many macromolecules in cytosolic volumes [36], [47], [48]. These models have been used to answer questions about macromolecular diffusion and stability outside of the scope of these atomistic models. However, it is possible that effects owing to metabolites could be important in these types of model.


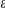
 of the cytosol of *E. coli* has many competing factors. Interestingly, total 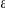 for the 50 M and tip3p systems are similar as the metabolite increment cancels the decrement of the monatomic ions of the tip3p+KCl 0.3M system. For the cytosol any increment in the rotational contribution due to proteins is an unknown and could have a large effect on 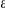, possibly only on a local level. Ubiquitin, used here, clearly has a large increment but can this be said of all proteins? A recent study has analyzed the dipole moments of the protein database [49] and gives an average protein biological unit dipole of 639 D, with ubiquitin having a dipole of 239 D. This suggests that most proteins have a dipole at least twice as big as ubiquitin. However, excluded volume will also have an effect reducing the effect of dipoles due to larger proteins. A higher dielectric compared to pure water will decrease electrostatic screening according to Debye-Huckel theory. A recent study has explored electrostatic screening using molecular dynamics and free energy calculations [50], and suggests that screening at high salt concentration is less than may have been expected from approximate treatments. Hence, the electrostatic screening found in cytosol solution may need further investigation. For the purposes of bio-simulations using implicit solvent it may be that a value closer to the 148 found here will give conditions closer to those found *in vivo*.

Owing to the diffusive and electrostatic considerations discussed above, it may be possible that proteins have a specific electrostatic and diffusive spheres of influence. If some proteins attract more metabolite ions than others, then this will again affect the local screening of the solution. Hence, proteins may have a locally specific electrostatic environments and propensities for associated metabolites and NIMS. In one example the electrostatic field of a protein is suggested to attract and orient specific metabolites [51], another study suggests that electric fields related to function are very protein specific and conserved through protein families [49].

Recently, kinetic models of cellular metabolism have started to appear in the literature [52]. These studies often attempt to approximate the thermodynamic activity of metabolites through Debye-Huckel theory [53]. Considering the high level of interaction between metabolites found in this study, the use of theory based on infinite dilution may not be sufficient to give realistic thermodynamic activities for these models. A recent experimental study has performed enzyme-inhibitor assays with an *in vivo* like solution (300 mM potassium, 50 mM phosphate, 245 mM glutamate, 20 mM sodium, 2 mM free magnesium and 0.5 mM calcium, at a pH of 6.8) rather than a standard concentration of the inhibitor [23]. In the *in vivo* like solution some enzymes have capacities (Vmax) which are less than half those found in optimised conditions. The solutions used are far from the complexity of the real cytosol and so further investigation of more complex solutions may be required. In the future it may be possible to calculate accurate thermodynamic activities using free energy calculations. These ideas may have implications for drug discovery. For example drug candidates predicted to spend significant amounts of time in NIMS and unavailable for binding to enzymes may not be optimal.

The behaviour and effect on the cytosol environment of molecules used by the cell to protect against stresses such as high osmolarity, pressure or anhydrobiotic conditions could be explored with simulations such as those in this study. A molecule which diffuses rapidly and is generally free from NIMS will be more osmotically active, if this molecule does not affect other aspects of the environment, would be a suitable osmolyte protectant. From this study we can predict that 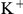, glyceric acid, malate, 3-phosphoglycerate, and phenolpyruvate (metabolite codes are in supporting information Table S1) may be more osmotically active than other metabolites of a similar size. These models represent a specific phase in the cell cycle in optimal external conditions. The constituents of the cytosol can change in response to many factors and inevitably properties such as diffusion rates and molecular associations can be effected. Additionally, understanding the effects of different metabolites, compatible solutes, osmolytes and ions on the properties of the cytosol may allow us to better understand the reactions of the cell to extreme environments such as high salt concentration, high temperature or desiccation [54].

The simulations carried out in this study give an interesting picture of the molecular behavior in the cytosol solution. Metabolites and proteins are seen to have significant level of non-ideal behavior, with metabolites forming large non-covalently interacting metabolite structures (NIMS) and proteins slowing the diffusivity of metabolites. The electrostatic fields of proteins are powerful and control the local dielectric conditions possibly allowing selective filtering of metabolites. In the future these types of simulations may, as part of comparative or thermodynamic analyses, shed light on many poorly understood aspects of cellular environments.

## Methods

### Theory

Molecular diffusion coefficients were calculated using the Einstein relation [55],
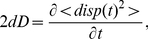
(1)where 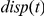 is the displacement of the atoms of a molecule over time 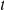, 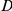 is the diffusion coefficient and 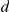 is the number of dimensions of the position data. The Einstein relation was chosen over the velocity correlation function owing to better convergence behavior and the lack of a need to store velocity data. Mean squared displacement (msd) plots were averaged over replicas of the data with 50 ps removed from the start of each successive replica and the linear regression was applied from 1000 to 3000 ps.

Solvent accessible surface area (SASA) was employed to show the amount of time each molecule spends free in solution or as part of a larger non-covalently interacting structures. SASA was calculated, using the “Double Cube Lattice Method” of Eisenhaber *et. al.* [56], for each molecule with and without the surrounding environment and the difference taken in order that the average molecular surface area in contact with other non-water molecules is found (average contact area). This average contact area was then displayed as a percentage of the average SASA of the metabolite or residue without the surrounding environment, the percentage contact area.

Another analysis calculates percentage of simulation which metabolites are in contact with other non-water molecules. Here only a thermodynamically significant contact was of interest. The average excluded SASA found when two hydrogen bonds were present for all metabolites was calculated from the 100 M simulation. Hence, here contact was defined by an excluded SASA threshold of 0.48 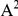. The use of SASA to define this contact means that other types of interaction such as those involving 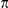 clouds are also included.

The calculation of 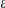 using computer simulation was originally reported by Neumann and Steinhauser [57]. The dielectric constant of water models in molecular mechanics simulations has often been calculated in the literature [58], [59]. These studies generally calculate the static dielectric constant 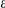 via the fluctuations of the system dipole 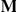,
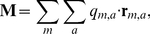
(2)

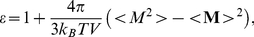
(3)where 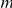 represents molecules and 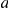 atoms in a molecule, 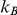 is the Boltzmann constant, 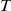 is the temperature and 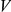 is the volume. 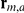 is generally the origin of the coordinate system or the center of mass of the system.

In the present study the use of equation 3 is difficult due to the presence of molecules with net charge. For a charged molecule the choice of reference position 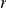 directly affects the molecular dipole. For an overall neutral system these differences are thought to cancel, however convergence can be extremely slow [60]. A recently developed methodology decomposes 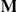 into rotational (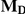) and translational (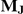) contributions [61],
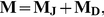
(4)

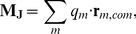
(5)

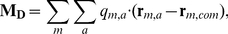
(6)where 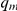 is the total charge of a molecule and 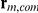 is the center of mass of a molecule. 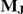 describes the position of charge centers through the system and 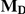 is the sum of molecular dipoles with respect to their center of mass. Combining equations 3 and 4 gives an equation for 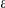 which may overcome some of the problems of equation 3 alone,
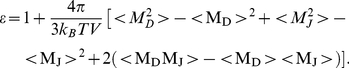
(7)


This can be further simplified this by assuming that we use enough data such that 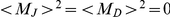 giving,
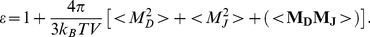
(8)


For convenience the rotational, translational and cross term contributions to 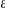 are denoted 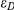, 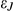 and 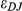 respectively with, 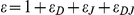. 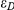 is calculated through a simple ensemble average of 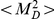. 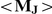 is directly related to the electrical current (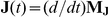) and therefore the static conductivity,
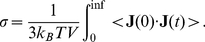
(9)


This means there are possible alternative routes to finding 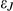 as 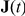 is also easily obtainable from molecular simulation. These possibilities have recently been explored in the case of simple ionic liquids [60], [62], [63]. Hence, in the present study 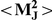 is found using the Einstein-Helfand method, as
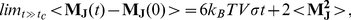
(10)where 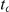 is the correlation length of current auto-correlation function. A linear regression fit of the resulting curve gives the static conductivity 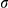 from the slope and 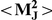 from the y-axis intercept. The cross term 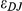 is certain to be very small. Recent studies have evaluated 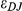 for a series of ionic liquids made up of molecules which all have both translational and rotational dipoles [62], [63]. All of these studies have found very small 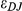. In the present study, a very small minority of molecules have both a translational and rotational dipoles, hence 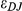 will be very small and has not been calculated.

### Simulation methods

All simulations used the GROMACS MD package [64], the OPLS force-field [65] was used for Zwitterionic protein residues and parameters for non-standard molecules were generated using hetgrpffgen provided with the Schrödinger Suite (Schrödinger LLC). This parameter generation method has recently been explored using solvation free energies of small, neutral molecules and was generally found to be of a high quality [66]. The development of the OPLSAA force field has focused on reproducing experimental measurements of thermodynamic properties for representative small molecules and was recently found to be the best at reproducing geometries and energies of inter-molecular complexes along with MMFF [45]. The recently developed Bussi *et. al.* thermostat was used, owing to its good reproduction of real dynamics and diffusive properties [67], [68]. The Parrinello-Rahman barostat was used for all production calculations. Temperature was set to 37 degrees Celsius.

Equation 8 must be applied to a periodic simulation using a long range electrostatic lattice summation and conducting boundary conditions, therefore periodic boundaries and particle mesh Ewald [69] was used throughout this study. Coulombic cutoffs at 1 nm have been shown to give more accurate dielectric calculations and were used throughout this study [57]. Lennard-Jones interactions were truncated with a switching function from 0.8 to 0.9 nm. System configurations were stored every 4 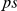 for the longer, 200 ns simulations. Subsequently, shorter 100 ns simulations were carried out storing configurations every 10 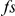 for the 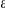 analysis.

Two box sizes were used, with dimensions of 50 Å and 100 Å, to assess possible size effects and provide a more tractable simulation for the 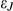 analysis. The numbers of metabolite molecules used in each box was calculated from concentrations measured by Bennett *et. al.* [24]. Metabolites with concentrations sufficiently low such that less than 0.5 metabolites would be found in a particular box size were not automatically included. However, the total observed intracellular metabolite concentration given by Bennett *et. al.* was 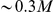. This total is a higher concentration than that found through automatically included metabolites (0.23 M). We chose to increase the total metabolite concentration to 0.28 M, by randomly selecting from a list of less abundant metabolites with a probability biased by their concentration.

It is not possible to accurately estimate from published metabolomics data the concentrations of free metabolities as opposed to the total metabolite concentration. However, particularly for the most abundant species, Bennett *et. al.* [24] suggest that the concentrations are well in excess of the Km of enzymes that consume the metabolites, ensuring saturation of the enzymes (which will generally have much lower concentrations), and suggesting that a significant portion of the high-concentration metabolites will be free in solution. Nonetheless, the concentrations we use may overestimate the free concentrations of the various metabolites to unknown and variable extents, which is a limitation of the current study.

All metabolites were protonated according to pKas at pH 7.6 [70] found either though experimental data or calculation with Epik (Schrödinger LLC). The methods used by Bennett *et. al.* were not able to detect putrescine (JD Rabinowitz, personal communication, 2010). Putrescine has a 2+ charge at pH 7.6 and thus was used to give a neutralising charge along with potassium and magnesium ions (magnesium was used to represent all 2+ mono-atomic ions). Concentrations of putrescine (28 mM), magnesium (40 mM) and potassium (290 mM) ions in line with literature studies [71]–[78] were added such that the system was neutralised. Putrescine and magnesium are often found interacting with DNA, RNA and other large macromolecules [79]–[81] and therefore are less likely to be found free in the cytosol and in our simulation boxes. While potassium may be more likely to be found free in the cytosol and is more osmotically active [82]–[84]. Hence, the amount of potassium ions should be more related to the osmotic strength of the external medium compared to other ions or metabolites.

Larger macromolecules (proteins) were also considered, and to this end 50 and 100 Å boxes containing ubiquitin were also constructed. Ubiquitin (PDB code 1UBQ) is a eukaryotic protein, it was chosen owing to its small size and large amount of literature dedicated to its study [25]–[27]. A protein concentration of 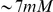 was assumed along with possible protein volume of 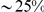 [5], [76], [85]. Table 1 shows the details of the four simulation boxes created for this study.

The effective concentration of the single ubiquitin in the 50 Å is around 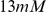 which is higher than desired, however making this box larger would have prohibited running simulations long enough for the 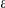 analysis. 50 Å boxes of tip3p water and tip3p with 0.3 M KCl (tip3p+KCl) were also created and equilibrated as part of the dielectric analysis. Types and numbers of metabolites used for each box are listed in supporting information, Table S1.

Model cytosol boxes were constructed through a simple Monte Carlo procedure. Each metabolite to be added to a box was treated as a buffered sphere and random positions were trialled until one was found which did not clash with the edge of the box or any other metabolite. Consequently, the initial structure of the boxes had no contact between any of the constituent metabolites. Owing to these considerations structural equilibration of the boxes was closely monitored before any analysis could be carried out. The use of a barostat throughout the structural equilibration is essential as the actual size of the simulation box reduces slightly.

## Acknowledgments

The authors thanks Dr. Andrew Cossins and Dr. Olga Vasieva for useful discussions over the biological issues discussed in this work.

## Supporting Information

Figure S1Metabolite structures found in the 100 Å boxes. Panal A is a structure stabilised by 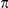 stacking from a 100 Å simulation without mg2+ ions. A structure stabilised by 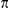 stacking between purine type groups. Panel B is a large NIMS stabilised by many mg2+ ions. mg2+ ions are enlarge pink blobs. Panel C is a small NIMS stabilised by four metabolites in a 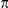 stacking formation. Panel D is two ubiquitin molecules in close contact with the charged patch of one interacting with the other. This view has been clipped in the far distance for clarity. mg2+ ions are depicted as large pink spheres.(TIFF)

Figure S2Bar-plot of average and maximum time of (A and C) contact and (B and D) full solvation events for all metabolites of the 100M (A and B) and 100U (C and D) simulation. Metabolites are listed in order of the number of atoms which they contain. A contact event is defined as a time period (consecutive frames of MD with frames every 4 ps) where the SASA excluded by other metabolites is greater than 0.48 Å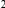 (see methods section). Conversely a full solvation event is a time period where the SASA excluded by other metabolites is less than than 0.48 Å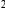. It seems that in general the smaller molecules spend more time free in the solvent than larger molecules. From this data we can build a picture of the behaviour of individual molecules. For example arginine (ARG) spends almost all of its time in contact with other metabolites further to this it probably is generally part of large, long lasting NIMS as its average and maximum contact event is very high. Of course this is no surprise as in these simulations ARG is one of only a few positively charged metabolites. Glyceric acid (GCC) seems to spend most of its time free in solution and its contact events are generally very short, suggesting it diffuses very quickly, momentarily interacting with many different entities.(TIFF)

Figure S3 A stacking NIMS remeniscent of the RNA polymerase elongation complex from a 100 Å simulation.
(TIFF)

Figure S4 Average contact area for the 100M against 100MP simulations for all types of metabolite. The red line represents contact area equality between 100M and 100MP simulations. The green and blue lines show the points at which, the 100MP and 100M simulations respectively, have 50% more contact area. NDP, ADP, CAP, PRP and CEA denote data points for NADPH, Adenosine Diphophate, Carbamylaspartate, Phosphoribosyl pyrophosphate, Co enzyme A-sh respectively.
(EPS)

Figure S5A comparison of percentage time which each metabolite is in contact with any another for the 100M and 100MP simulations. Contact is defined through SASA contact area of 0.48 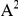 (see methods section).(EPS)

Figure S6 Plot of 50M against 100M diffusion coefficients. Values were calculated from regression analysis of 100 ps to 2000 ps of a msd plot restarted every 50 ps. Standard errors were calculated from analysis of multiple copies of the same metabolite, metabolites represented only once do not have errors.
(EPS)

Figure S7 Plot of diffusion coefficients for glutamate in successive regions of the 100M simulation. Values were calculated from regression analysis of 100 ps to 2000 ps of a msd plot restarted every 50 ps. Standard errors were calculated from analysis of multiple copies of the same metabolite.
(EPS)

Figure S8Einstein-Helfand plot of the MSD of 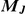 for 50M, 50MP and the tip3p + KCL systems. These plots were scaled by 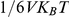 to aid comparison to 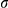 values of Table 2 in the main document. Y-axis intercept of these plots is equal to 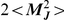.(EPS)

Table S1 Numbers of metabolites used in each cytoplasm simulation box. Types and numbers of metabolites used in each cytoplasm simulation box along with percentage contact time (as defined in the methods section) for 100M and 100MP simulations. The percentage contact time is also presented in Figure 5 of the main document. Also, diffusion coefficients (D) are presented to complement Figure 7 of the main document.
(TIFF)

Table S2 Percentage SASA contact area for residues of ubiqitin. Percentage SASA contact for residues of ubiqitin averaged over the four ubiquitin molecules of the 100U simulation. Data is arranged in order of percentage SASA contact.
(TIFF)
