# Supplementary figures and images for: A New View of the Bacterial Cytosol Environment
Source: PLoS Comput Biol. 2011 Jun 9;7(6):e1002066. doi: 10.1371/journal.pcbi.1002066 (PMC3111478; doi:10.1371/journal.pcbi.1002066)

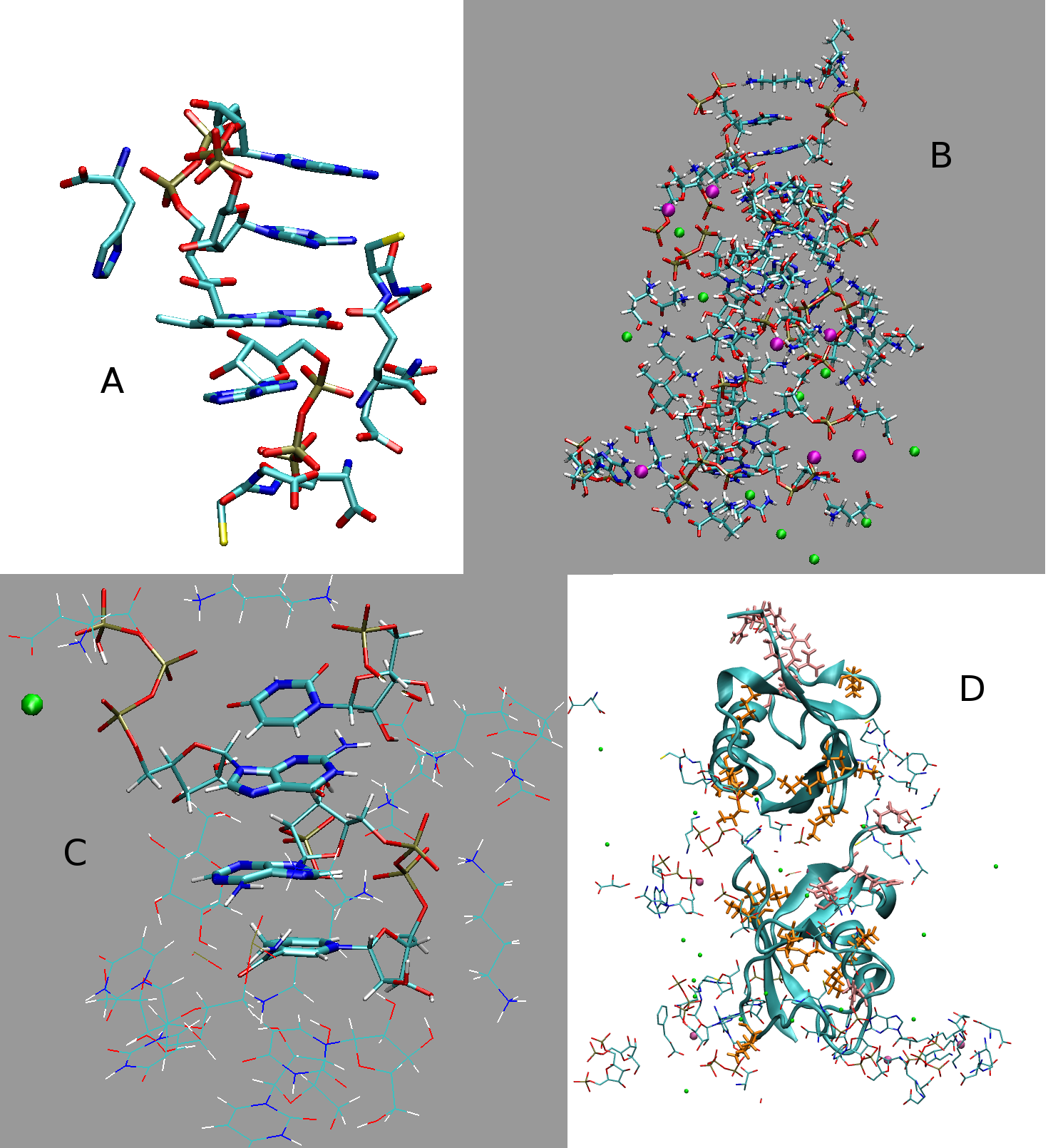

Supplement: Figure S1 — Metabolite structures found in the 100 Å boxes. Panal A is a structure stabilised by stacking from a 100 Å simulation without mg2+ ions. A structure stabilised by stacking between purine type groups. Panel B is a large NIMS stabilised by many mg2+ ions. mg2+ ions are enlarge pink blobs. Panel C is a small NIMS stabilised by four metabolites in a stacking formation. Panel D is two ubiquitin molecules in close contact with the charged patch of one interacting with the other. This view has been clipped in the far distance for clarity. mg2+ ions are depicted as large pink spheres. (TIFF) [file pcbi.1002066.s001.tif]

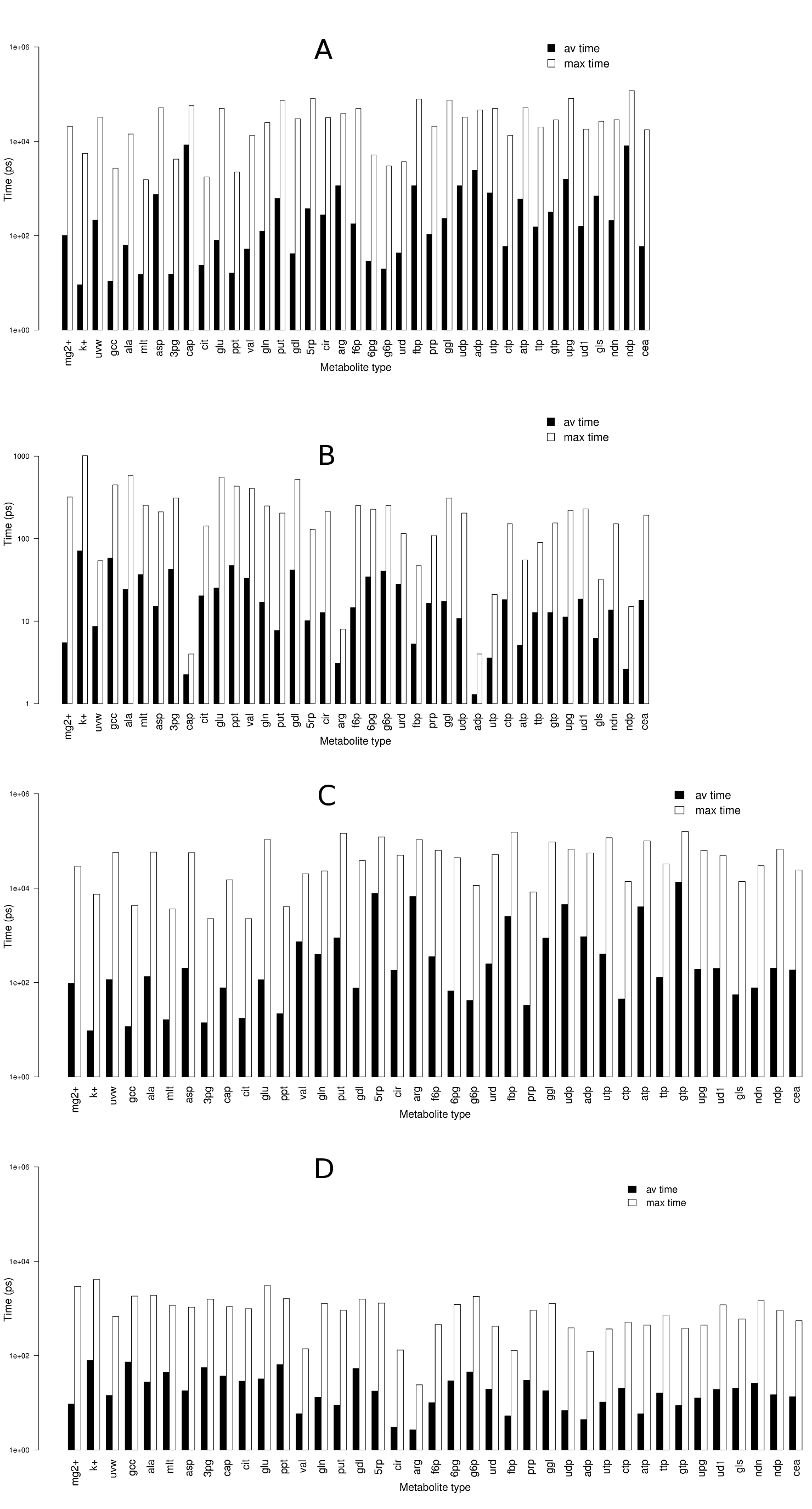

Supplement: Figure S2 — Bar-plot of average and maximum time of (A and C) contact and (B and D) full solvation events for all metabolites of the 100M (A and B) and 100U (C and D) simulation. Metabolites are listed in order of the number of atoms which they contain. A contact event is defined as a time period (consecutive frames of MD with frames every 4 ps) where the SASA excluded by other metabolites is greater than 0.48 Å (see methods section). Conversely a full solvation event is a time period where the SASA excluded by other metabolites is less than than 0.48 Å. It seems that in general the smaller molecules spend more time free in the solvent than larger molecules. From this data we can build a picture of the behaviour of individual molecules. For example arginine (ARG) spends almost all of its time in contact with other metabolites further to this it probably is generally part of large, long lasting NIMS as its average and maximum contact event is very high. Of course this is no surprise as in these simulations ARG is one of only a few positively charged metabolites. Glyceric acid (GCC) seems to spend most of its time free in solution and its contact events are generally very short, suggesting it diffuses very quickly, momentarily interacting with many different entities. (TIFF) [file pcbi.1002066.s002.tif]

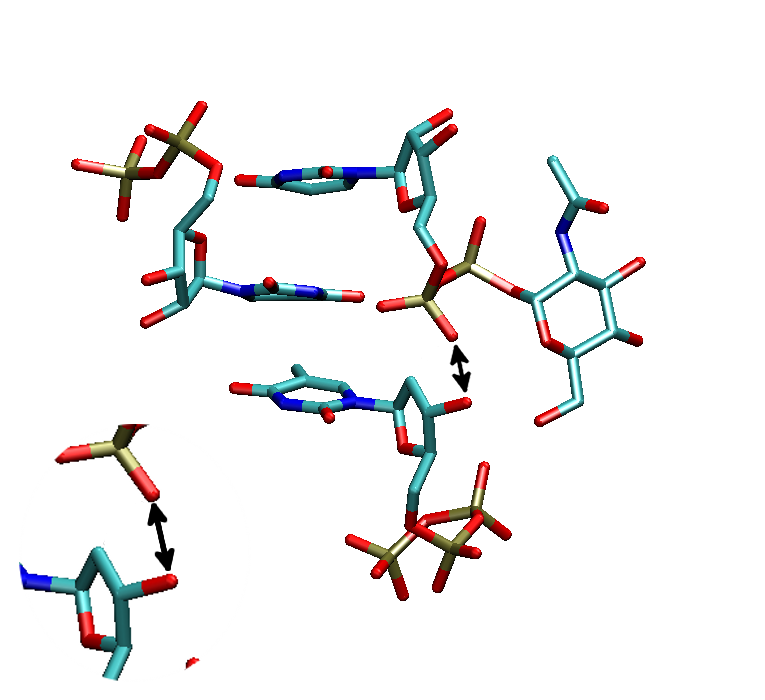

Supplement: Figure S3 — A stacking NIMS remeniscent of the RNA polymerase elongation complex from a 100 Å simulation. (TIFF) [file pcbi.1002066.s003.tif]

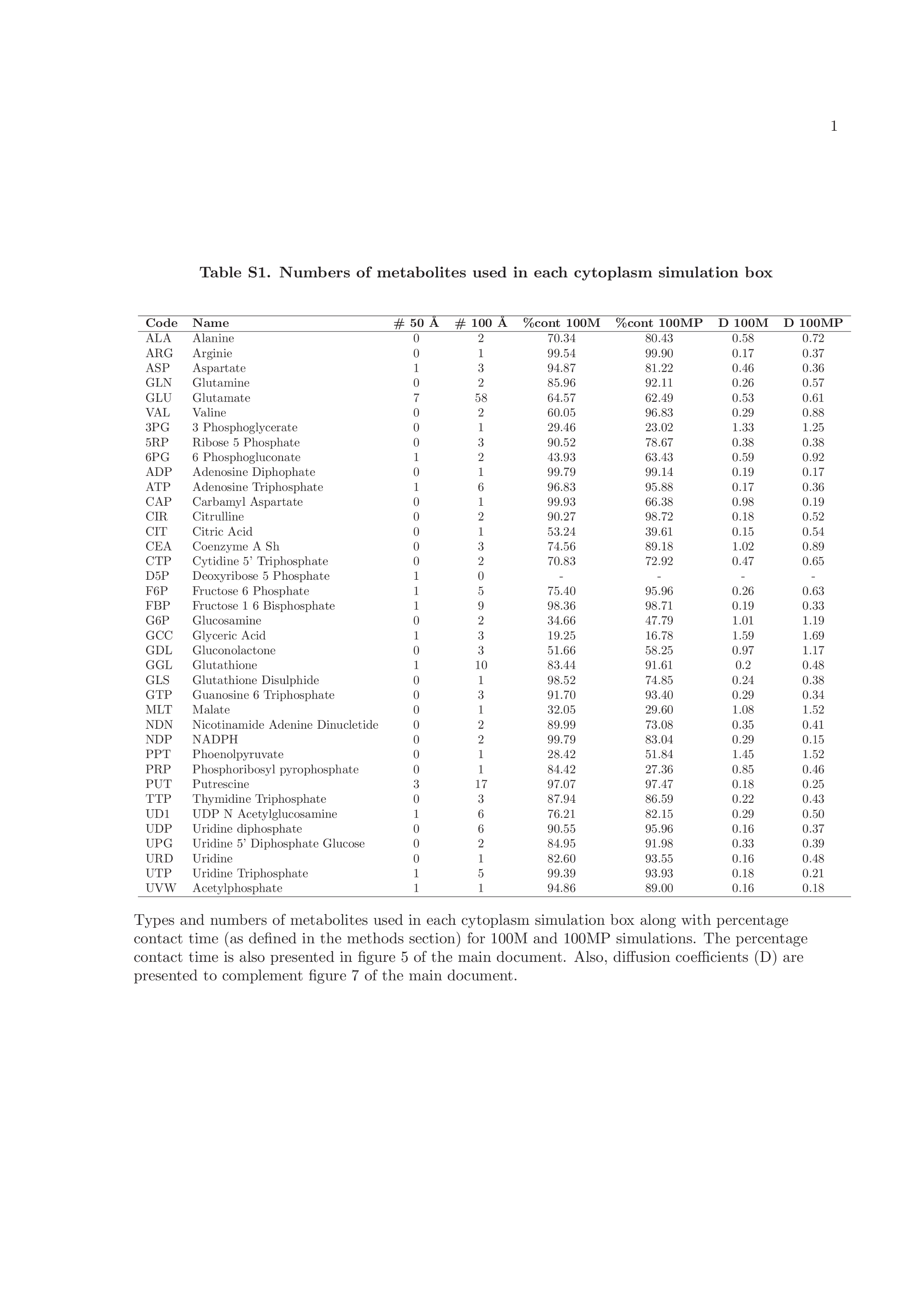

Supplement: Table S1 — Numbers of metabolites used in each cytoplasm simulation box. Types and numbers of metabolites used in each cytoplasm simulation box along with percentage contact time (as defined in the methods section) for 100M and 100MP simulations. The percentage contact time is also presented in Figure 5 of the main document. Also, diffusion coefficients (D) are presented to complement Figure 7 of the main document. (TIFF) [file pcbi.1002066.s009.tiff]

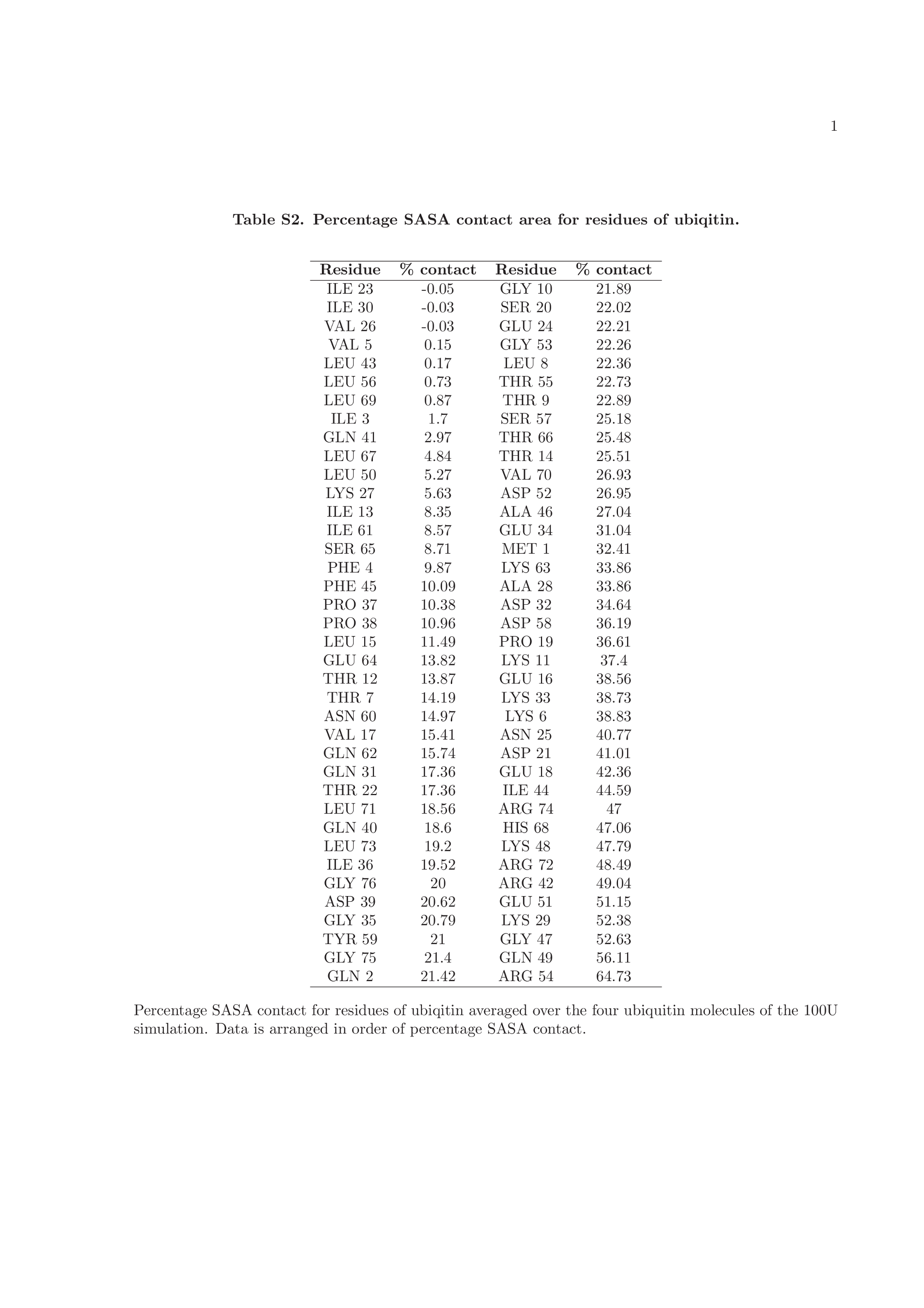

Supplement: Table S2 — Percentage SASA contact area for residues of ubiqitin. Percentage SASA contact for residues of ubiqitin averaged over the four ubiquitin molecules of the 100U simulation. Data is arranged in order of percentage SASA contact. (TIFF) [file pcbi.1002066.s010.tiff]
